# Hypotony following the use of topical carbonic anhydrase inhibitors in glaucoma patients: a case report and review of literature

**DOI:** 10.1097/RC9.0000000000000457

**Published:** 2026-04-21

**Authors:** Sulaiman M. AlTariqi, Mohammed A. Halawani, Nayef D. AlOtaiby

**Affiliations:** Glaucoma Division, Department of Ophthalmology, King Khaled Eye Specialist Hospital, Riyadh, Saudi Arabia

**Keywords:** carbonic anhydrase inhibitors, case report, dorzolamide, filtering glaucoma surgery, glaucoma, ocular hypotony

## Abstract

**Introduction and importance:**

Topical carbonic anhydrase inhibitors (CAIs) are widely used to lower intraocular pressure (IOP) by suppressing aqueous production. Though generally safe, hypotony is an extremely rare adverse effect. Understanding, this reaction is clinically relevant, particularly in surgically complex glaucoma eyes.

**Case presentation:**

We report a monocular 70-year-old patient with congenital glaucoma who developed marked hypotony shortly after initiating topical dorzolamide 2%. The patient presented with blurry vision and ocular discomfort, with examination showing an IOP of 4 mm Hg measured using Goldmann applanation tonometry. Ancillary imaging ruled out cyclodialysis cleft and choroidal or retinal pathology.

**Clinical discussion:**

Dorzolamide was immediately discontinued, resulting in the complete recovery of IOP to 18 mm Hg within 2 weeks, while other antiglaucoma medications were continued unchanged. Subsequent exposure to other CAIs resulted in similar hypotensive responses, supporting a medication-related mechanism. No hypotony maculopathy or structural complications developed.

**Conclusion:**

This case underscores the importance of considering CAI-induced hypotony in patients with prior glaucoma surgeries or prolonged low IOP. Early recognition and prompt medication withdrawal can prevent permanent structural damage and preserve vision. The accompanying literature review highlights CAIs, particularly dorzolamide, as a rare but significant cause of hypotony.

## Introduction

Glaucoma is the second leading cause of blindness and the most common cause of irreversible blindness worldwide^[^1^]^. It is characterized by progressive irreversible optic neuropathy with associated characteristic visual field changes and elevated intraocular pressure (IOP) as a main modifiable risk factor. Medical and/or surgical management are used to control disease progression depending on the type and stage of glaucoma. IOP control can be achieved medically by using pharmacological compounds that decrease aqueous humor production or increase ocular aqueous outflow. Carbonic anhydrase inhibitors (CAIs) are commonly used either systemically or topically for managing IOP. Topical CAIs reduce IOP by inhibiting carbonic anhydrase isoenzyme II in the non-pigmented ciliary epithelium, leading to reduced bicarbonate formation and subsequent suppression of aqueous humor secretion^[^2^]^. Common side effects of CAI include ocular discomfort, blurry vision, corneal punctate erosions, and corneal endothelial damage^[^3^]^. Hypotony is an extremely rare complication reported with the use of topical CAI. A review of the literature reveals fewer than 10 reported cases of ocular hypotony associated with topical CAIs, most occurring in eyes with prior glaucoma surgery^[^4–7^]^. In this report, we present a case of a patient who developed hypotony following the use of topical and systemic CAI and review the current literature on similar cases. This case report has been reported in line with the SCARE 2025 guidelines^[^8^]^.

HIGHLIGHTS

- Hypotony developed following the use of topical carbonic anhydrase inhibitors in a glaucoma patient with prior filtering surgeries.
- Intraocular pressure normalized rapidly after discontinuation of the CAI, supporting a medication-related etiology.
- Review of the literature shows that most reported cases occurred in eyes with previous glaucoma surgery.
- Dorzolamide is the most frequently implicated aqueous suppressant associated with hypotony.
- Clinicians should monitor high-risk patients closely when initiating CAIs, especially those with prolonged low IOP or prior filtration procedures.

## Case presentation

**Figure 1.**
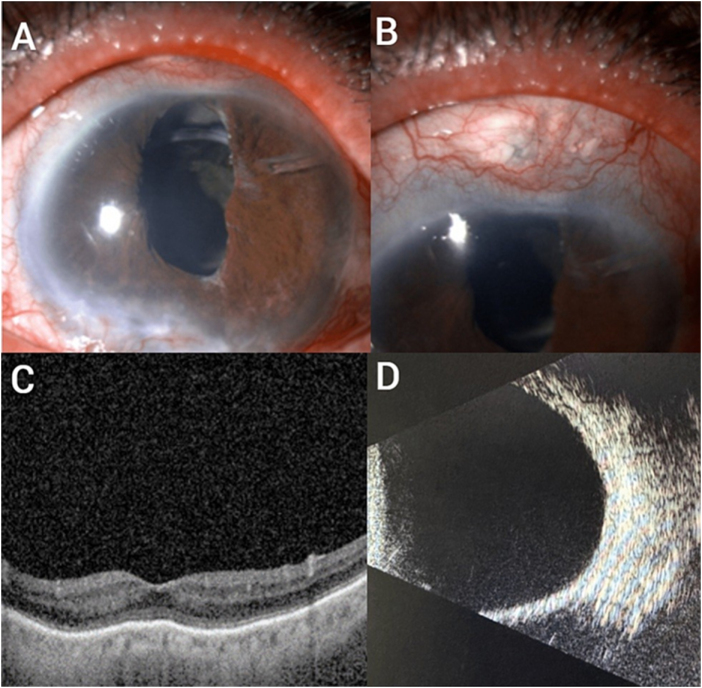
Slit-lamp photos of the anterior segment, optical coherence tomography macula, and B-scan ultrasound.

A 70-year-old monocular male with congenital glaucoma presented to the glaucoma clinic complaining about mild pain and blurry vision in the left eye that occurred gradually with no history of trauma or recent surgical intervention. The patient was recently started on topical dorzolamide 2% to decrease IOP to a target pressure despite being on brimonidine 0.15%, timolol 0.5%, and latanoprost 0.005% with good compliance. The patient had been on these medications for a long term with stable IOP prior to the addition of dorzolamide. He had previously undergone trabeculectomy with adjunctive mitomycin C, phacoemulsification with endoscopic cyclophotocoagulation, and Ahmed glaucoma valve implantation in the left eye. The patient had a prosthesis in the right eye following evisceration due to childhood trauma. On presentation, best-corrected visual acuity was 20/400, and IOP was 4 mm Hg. IOP was consistently measured using Goldmann applanation tonometry at all visits. Central corneal thickness measured 538 µm in the affected eye and 542 µm in the fellow eye. Slit-lamp examination showed a quiet anterior segment with a scarred bleb, stable and well-covered glaucoma valve, inferior corneal scar with pannus, deep anterior chamber, centered and well-positioned posterior chamber intraocular lens, advanced cup-to-disc ratio, and flat retina (Fig. 1). Gonioscopy indicated an open angle with no signs of a cyclodialysis cleft and a stable paracentral small island on the visual field. B-scan ultrasonography demonstrated a flat retina without choroidal detachment or effusion (Fig. 1). There were no clinical signs of hypotony maculopathy, and optical coherence tomography of the macula showed no macular folds or subretinal fluid (Fig. 1). Prior to dorzolamide initiation, IOP ranged between 14 and 16 mm Hg; following initiation, IOP decreased to 4 mm Hg and recovered to 18 mm Hg within 2 weeks after discontinuation. The provisional diagnosis was medication-induced hypotony with unknown mechanisms. The diagnosis was based on low IOP associated with visual symptoms rather than isolated IOP reduction. Subsequent exposure to other CAIs resulted in similar hypotensive effects.

(A) Slit-lamp photo of the anterior segment with diffuse illumination showing normal lids, mild conjunctival injection, peripheral corneal pannus inferiorly, clear central cornea, superior temporal tube in good position and posterior chamber intraocular lens. (B) Closer look at the filtrating bleb area showed vascularized low bleb. (C) Optical coherence tomography of the macula showing intact retinal layers with no macular folds or subretinal/choroidal fluid. (D) B-scan ultrasonography showed a flat retina and no choroidal detachment.

## Clinical discussion

The World Glaucoma Association defines ocular hypotony as an IOP of less than 5 mm Hg, particularly when associated with clinical signs or visual dysfunction^[^9^]^. Hypotony can be caused by excessive aqueous drainage as in the cases of cyclodialysis cleft, rhegmatogenous retinal detachment, choroidal detachment, and glaucoma surgeries with overfiltration. However, decreased aqueous production can lead to hypotony as in the cases of ciliary body (CB) hypoperfusion in ocular ischemic syndrome, CB detachment in acute or chronic uveitis, and due to aqueous suppressant medications, which are considered very rare^[^10^]^. Before making a presumptive diagnosis of medication-induced hypotony, it is imperative to rule out the other causes. In 1985, Vela *et al* published the first report in the literature of four patients who developed ocular hypotony following initiation of aqueous suppressants in the form of timolol and acetazolamide^[^6^]^. Three of these patients had previously undergone glaucoma filtering surgery (Table 1)^[^6^]^. This observation was supported by two reports by Fineman *et al* and Sharma *et al* who described three cases with ocular hypotony who had previous glaucoma filtering surgery following initiation of topical dorzolamide 2% (Table 1)^[^5,7^]^. Notably, hypotony has been reported with several aqueous suppressants, with dorzolamide 2% being the most frequently implicated. The diagnosis in most cases was ciliochoroidal detachment, which was not evident in the present case. Prolonged periods of low IOP – whether surgically or medically induced – could have sensitized the ciliary epithelium and led to a near-total reduction in aqueous production once the eye was challenged with an aqueous suppressant^[^6^]^. This can explain the fact that most of the affected eyes in the previous reports, along with the present case, have undergone previous glaucoma filtering procedures, specifically, trabeculectomy, which is known to significantly decrease IOP for prolonged periods postoperatively. The temporal relationship between dorzolamide initiation and the onset of hypotony, combined with prompt normalization of IOP after drug discontinuation while other medications were continued, strongly supports dorzolamide as the causative agent. Although prior glaucoma surgery may predispose eyes to exaggerated IOP reduction, the patient maintained stable IOP for several years postoperatively, making delayed surgical hypotony unlikely. An insight that could help in the diagnosis of medication-induced ocular hypotony is the rapid improvement of the clinical signs as soon as the offending medication is discontinued. Rechallenging the affected eye with a medication of a similar class usually results in an identical effect as shown in this report. Caution should be exercised, and closer follow-up is recommended when a medication of a similar mechanism of action is utilized, as it theoretically could have the same effect. A formal rechallenge with dorzolamide was not performed due to concern for recurrent hypotony and potential vision-threatening complications, which represents a limitation of this report.

**Table 1:** Summary of the reported cases in the literature.

Report	Cases	Type of glaucoma (*n*)	Medication	Previous glaucoma surgery (*n*)	Diagnosis (*n*)	Outcome following discontinuation of the medication (*n*)	Recurrence
Vela *et al* ^[^6^]^	4	POAG (3); not mentioned (1)	Timolol 0.5% (3); acetazolamide (3)	Trabeculectomy (3); posterior lip sclerectomy (1)	Ciliochoroidal detachment (4)	Resolved (3); required surgery (1)	None
Fineman *et al*^[^5^]^	2	Not mentioned (2)	Dorzolamides 2% (2)	Trabeculectomy (1); posterior lip sclerectomy (1)	Ciliochoroidal detachment (2)	Resolved (1); required surgery (1)	None
Davani *et al*^[^4^]^	1	Not mentioned	Dorzolamides 2%	None	Ciliochoroidal detachment	Resolved	None
Sharma *et al*^[^7^]^	1	POAG	Dorzolamides 2%; timolol 0.5%	Trabeculectomy	Ciliochoroidal detachment	Resolved	None
Current report	1	Primary congenital glaucoma (PCG)	Dorzolamides 2%	Trabeculectomy AGV	Non-specific	Resolved	None
Total	9	POAG (4); not mentioned (4); pCG (1)	Dorzolamides 2% (5); timolol 0.5% (4); acetazolamide (3)	Trabeculectomy (6); posterior lip sclerectomy (2); aGV (1)	Ciliochoroidal detachment (8); non-specific	Resolved (7); required Surgery (2)	None

POAG, primary open-angle glaucoma

## Conclusion

Medication-induced ocular hypotony is considered among the least common causes of ocular hypotony. A stepwise approach that involves ruling out other differential diagnoses and discontinuation of the offending medication can result in a favorable outcome. Early recognition of medication-induced ocular hypotony and prompt withdrawal of the offending agent can prevent permanent structural damage and preserve visual function.

## Data Availability

The data supporting the findings of this study are available from the corresponding author upon reasonable request.
